# Airport Exit and Entry Screening for Ebola — August–November 10, 2014

**Published:** 2014-12-12

**Authors:** Clive M. Brown, Aaron E. Aranas, Gabrielle A. Benenson, Gary Brunette, Marty Cetron, Tai-Ho Chen, Nicole J. Cohen, Pam Diaz, Yonat Haber, Christa R. Hale, Kelly Holton, Katrin Kohl, Amanda W. Lee, Gabriel J. Palumbo, Kate Pearson, Christina R. Phares, Francisco Alvarado-Ramy, Shah Roohi, Lisa D. Rotz, Jordan Tappero, Faith M. Washburn, James Watkins, Nicki Pesik

**Affiliations:** 1 Division of Global Migration and Quarantine, National Center for Emerging and Zoonotic Infectious Diseases, CDC; 2 Center for Surveillance, Epidemiology and Laboratory Services, CDC; 3 Division of Global Health Protection, Center for Global Health, CDC

In response to the largest recognized Ebola virus disease epidemic now occurring in West Africa, the governments of affected countries, CDC, the World Health Organization (WHO), and other international organizations have collaborated to implement strategies to control spread of the virus. One strategy recommended by WHO calls for countries with Ebola transmission to screen all persons exiting the country for “unexplained febrile illness consistent with potential Ebola infection.” Exit screening at points of departure is intended to reduce the likelihood of international spread of the virus. To initiate this strategy, CDC, WHO, and other global partners were invited by the ministries of health of Guinea, Liberia, and Sierra Leone to assist them in developing and implementing exit screening procedures. Since the program began in August 2014, an estimated 80,000 travelers, of whom approximately 12,000 were en route to the United States, have departed by air from the three countries with Ebola transmission. Procedures were implemented to deny boarding to ill travelers and persons who reported a high risk for exposure to Ebola; no international air traveler from these countries has been reported as symptomatic with Ebola during travel since these procedures were implemented.

On October 11, 2014, after the first imported Ebola case was identified in the United States, an enhanced U.S. entry screening program was started at five international airports as an added measure to identify travelers from the three countries with widespread Ebola transmission who might have been exposed to Ebola within 21 days before arrival or who currently had signs or symptoms of Ebola. Entry screening first began at John F. Kennedy International Airport (JFK) in New York City, then Newark Liberty International Airport (EWR), Washington-Dulles International Airport (IAD), Chicago O’Hare International Airport (ORD), and Hartsfield-Jackson Atlanta International Airport (ATL). This program also allowed federal authorities to educate travelers, obtain their contact information, and link them with state and local partners to facilitate health monitoring, as appropriate, and prompt referral for care if they became ill. Of 1,993 travelers screened during October 11–November 10, 86 (4.3%) were referred to CDC public health officers for additional evaluation, and seven (8.1%) of the 86 were symptomatic and referred for medical evaluation (Table 1). None of the seven were diagnosed with Ebola.

The 1,993 travelers arrived in the United States after transit in at least one other country and had final destinations in 46 states; the most common destinations were New York (19%), Maryland (12%), Pennsylvania (11%), Georgia (9%), and Virginia (7%) (Figure). Entry screening provided public health departments with contact information for travelers to facilitate monitoring and provided an added layer of protection for the U.S. public.

On August 8, 2014, the International Health Regulations Emergency Committee determined that the Ebola outbreak in West Africa met the conditions for a Public Health Emergency of International Concern (1). The committee advised that WHO member states with Ebola transmission “should conduct exit screening of all persons at international airports, seaports and major land crossings, for unexplained febrile illness consistent with potential Ebola infection.”

## Exit Screening in Three Countries Most Affected

To advise on exit screening and other border control measures, CDC deployed staff members to Liberia and Guinea, beginning August 4, and to Sierra Leone, beginning August 9. In response to the importation of a case of Ebola into Nigeria with subsequent spread among health care workers and in the community, a CDC team was deployed to Nigeria on August 11.

WHO recommends that exit screening consist of a health questionnaire, a temperature measurement, and, if there is a fever, an assessment of the likelihood of the fever being caused by Ebola (1). According to WHO recommendations, Ebola patients or contacts, or persons with an illness consistent with Ebola, should not be allowed to travel unless the travel is part of an appropriate medical evacuation.

CDC worked with in-country partners (e.g., ministries of health and airport authorities) to enhance exit screening procedures; recommendations were tailored to each country’s needs to address critical gaps identified in their exit screening processes and procedures. Activities included developing and delivering training on the signs and symptoms of Ebola, exit screening procedures and documentation, and appropriate use of personal protective equipment. CDC also worked to conduct train-the-trainer sessions to ensure that the exit screening activities in place could be sustained. To help countries with no Ebola transmission detect and manage Ebola cases at points of entry, CDC developed templates and materials that other countries could consider and adapt as needed.* To estimate the number of travelers to the United States from the three countries most affected by Ebola, CDC used flight data software from Diio LLC (Reston, Virginia).

During August–October 2014, approximately 80,000 travelers departed the three most affected countries (Guinea, Liberia, and Sierra Leone) by air; approximately 12,000 of these travelers were en route to the United States. Procedures were implemented to deny boarding to ill persons and persons reporting a high risk of exposure to Ebola. No traveler who was denied boarding for fever or other symptoms or reported exposures has been reported as diagnosed with Ebola. Of those who were permitted to travel, none are known to have had Ebola symptoms during travel and none have been subsequently diagnosed with Ebola. Two travelers to the United States, who were not symptomatic during exit screening and travel, became ill with Ebola after arrival.

## Enhanced Entry Screening in the United States

Since July 2014, CDC has enhanced its routine procedures for detecting ill travelers entering the United States at airports by providing additional guidance and training to partners, including U.S. Customs and Border Protection (CBP), which inspects all arriving travelers seeking admission into the United States, airlines, airport authorities, and emergency medical service units at airports; the training covers recognizing possible signs of Ebola in travelers and reporting suspected cases to CDC. On October 11, 2014, CDC, in partnership with CBP, further enhanced efforts to identify ill travelers and travelers possibly exposed to Ebola by initiating an additional screening measure for travelers arriving from Guinea, Liberia, or Sierra Leone. Although identification of ill travelers remains an important goal of U.S. entry screening, enhanced entry screening also has four broader objectives: 1) to identify, on their arrival in the United States, travelers who might be ill with Ebola or who might have had exposure to Ebola, 2) to ensure that these travelers are directed to medical care, if needed, 3) to provide travelers with information on reporting fever and other symptoms to public health authorities, and 4) to rapidly provide the travelers’ contact information to public health authorities for active or direct active monitoring.†

CDC and CBP began enhanced entry screening at JFK on October 11 and on October 16 at four other airports (EWR, IAD, ORD, and ATL). Together, the five airports are estimated to handle 94% of all travelers arriving in the United States who had been in Liberia, Sierra Leone, and Guinea within the previous 21 days. Six days later, the Department of Homeland Security exercised its authority to direct passengers flying from the three countries to arrive in the United States at one of the five airports with enhanced screening.

For each person arriving from one of these three countries, CBP provides health information developed by CDC that includes facts about Ebola, symptoms to look for and what to do if symptoms develop, a wallet card, and information for clinicians to manage travelers who seek medical attention. Each traveler also receives a digital thermometer§ and instructions on how to use it. Travelers are screened with questions about symptoms and potential exposure risks, temperature checks (using noncontact thermometers), and visual observation for other symptoms of Ebola. If travelers have self-reported or measured fever or other symptoms or have been in a situation where exposure to Ebola could have occurred, CBP officers refer them to CDC public health officers stationed at the airport for further evaluation. Public health risk assessment of travelers is based on CDC-issued guidance (2), which classifies potentially exposed persons into three risk categories, whether symptomatic or not: high risk, some risk, and low (but not zero) risk. The guidance provides recommendations for the monitoring of these persons and their safe movement to avoid potential exposure of others during commercial travel (e.g., by airplane, ship, train, or long-distance bus). CDC officers determine whether travelers can continue to their destinations, and whether transport to a hospital for medical evaluation of symptomatic travelers is needed. CDC coordinates disposition of symptomatic travelers and those in the high and some risk categories with the appropriate state health department at the time of assessment. Persons identified as at high risk for exposure to Ebola would not be allowed to travel further on public conveyances.

All travelers in the three risk categories are referred for continued monitoring and support to a local health department based on their travel destination. Contact information for travelers arriving from countries with widespread Ebola transmission is entered into a database and transmitted to states through CDC’s Epidemic Information Exchange (Epi-X), a secure notification system. Once the states receive the travelers’ contact information, public health authorities can initiate appropriate monitoring and movement restrictions based on risk.

Of the 1,993 arriving in the U.S. from countries with widespread Ebola transmission who have been screened, 85% were adults aged ≥18 years, and 3% had reportedly worked in a health care facility or laboratory in a country with widespread Ebola transmission. According to flight data, it is estimated that less than 0.06% of total arrivals to the U.S. arrive from the three countries.

Among the 1,993 screened travelers, 86 (4.3%) were referred to CDC public health officers; of these, seven (8.1%) were referred for medical evaluation (Table 2). Of the persons interviewed by CDC for evaluation, all 86 were health care workers, of whom 70 were determined to be in the low (but not zero) risk category (2); nine of the 86 were laboratory workers, all of whom were placed in the low (but not zero) risk category. Since entry screening started, no traveler has been placed in the high risk category; one person became symptomatic after travel and was diagnosed with Ebola 6 days after arrival in the United States.

As of November 26, 2014, approximately 15,900 cases of Ebola have been reported by WHO. Of the four cases reported by the United States, two were among travelers from countries with widespread Ebola transmission. The first of these was a traveler from Liberia (3) who had no fever or declared symptoms or exposures at his exit screening in Liberia, was not symptomatic during travel, and developed symptoms after arrival in the United States. The second travel-related case was in a health care worker who returned from Guinea and who did not have symptoms during exit screening at departure, during travel, or at entry screening on arrival in the United States before developing Ebola.

### Discussion

Effective exit screening procedures in countries with widespread transmission of Ebola helped instill confidence that persons symptomatic with Ebola would be unlikely to travel. Humanitarian assistance is vital for combating the Ebola epidemic and reducing the risk for the disease being exported.

Airport exit or entry screening might not identify asymptomatic infected persons without recognized or declared exposures (4). Screening of travelers at departure from countries with widespread Ebola transmission and upon arrival in the United States is part of a comprehensive and layered strategy to protect travelers and U.S. communities and also includes 1) communicating to the traveling public by way of travel health alerts and other travel guidance posted online (at http://www.cdc.gov/travel), 2) denial of boarding to ill persons before travel, 3) reporting of persons who become ill onboard U.S.-bound airlines, and 4) monitoring for 21 days after the last possible exposure of persons from countries with widespread Ebola transmission, based on their exposure risk category by U.S. public health authorities.

On November 17, 2014, CDC and CBP also began screening for Ebola travelers from the West African nation of Mali upon entry to the United States after reports of confirmed cases in that country. In the United States, entry screening enables public health authorities to identify persons arriving from countries with widespread Ebola transmission and provide them with public health guidance about how to monitor themselves for symptoms of Ebola, as well as the tools with which to monitor themselves, links to public health authorities, and information needed to contact public health or medical authorities if they develop a fever or other symptoms.

Together, the combined exit and entry screening processes achieve the following six outcomes; they: 1) prevent travel by ill persons from countries with widespread Ebola transmission until they have had appropriate medical evaluation, 2) reduce the likelihood of a traveler from a country with widespread Ebola transmission becoming ill during travel, 3) allow the quick identification of any illness in persons arriving from countries with widespread Ebola transmission, 4) limit contact of persons being evaluated for Ebola with other persons, 5) facilitate rapid and appropriate clinical care for ill travelers, and 6) provide the arriving traveler with public health education and links with public health authorities.

Although the magnitude of the current Ebola outbreak in West Africa has challenged established approaches, isolation of cases and contact tracing remain essential to contain the disease and prevent spread to other counties. Outbreak responses to severe acute respiratory syndrome (5), 2009 pandemic influenza A(H1N1) (6,7) and Middle East respiratory syndrome-coronavirus (8) have demonstrated that, in an increasingly connected world, no destination is safe from the importation of emerging pathogens as long as pathogens are spreading anywhere in the world.

**What is already known on this topic?**

The World Health Organization (WHO) recommends that countries with Ebola transmission screen all persons exiting the country for febrile illness consistent with potential Ebola infection. WHO recommends that exit screening consist of a health questionnaire, a temperature measurement, and, if there is a fever, an assessment of the likelihood of the fever being caused by Ebola. According to WHO recommendations, Ebola patients or contacts, or persons with an illness consistent with Ebola, should not be allowed to travel unless the travel is part of an appropriate medical evacuation.

**What is added by this report?**

This report describes results of the use of exit and entry screening processes as part of a comprehensive strategy to reduce the likelihood that symptomatic travelers board commercial flights and cause transmission of Ebola. To date, there has been no indication of a risk for Ebola disease transmission related to international air travel. Of the 1,993 persons screened for Ebola after arriving in the United States from Guinea, Liberia, and Sierra Leone, none were symptomatic during travel. A total of 86 were referred to CDC public health officers for additional evaluation, and seven of the 86 were found to be symptomatic and referred for medical evaluation; none had Ebola.

**What are the implications for public health practice?**

These processes help to maintain confidence that air travel is safe from Ebola, identify potentially ill or exposed travelers, educate and inform the traveler, link the traveler with public health authorities for the duration of the incubation period, and facilitate the rapid detection of illness and implementation of appropriate public health control measures. State and local public health authorities are provided with timely information on arrivals from countries with widespread Ebola transmission to facilitate active or direct active monitoring based on travelers’ risk categorizations.

CDC has worked with international partners to establish and strengthen exit screening at ports of departure in-country and with domestic partners to conduct entry screening upon arrival into the United States. The goal and potential benefit of exit and entry screening at international borders encompasses more than identification of ill travelers at those borders. Using these processes to educate each traveler and then link the traveler to public health authorities for the duration of the incubation period is of critical importance to facilitate rapid detection of illness and implementation of appropriate public health control measures.

## Figures and Tables

**FIGURE:**
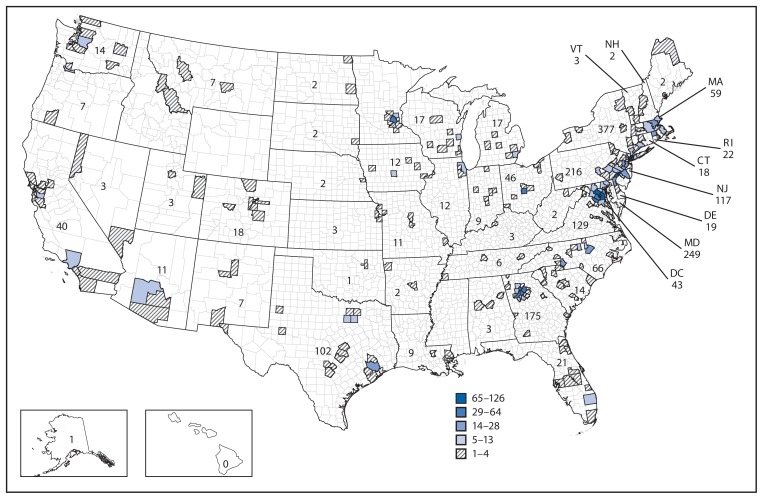
Number of travelers (N = 1,986^*^) arriving from Guinea, Liberia, and Sierra Leone who were screened for Ebola at U.S. airports, by state and county of destination — October 11–November 10, 2014 **Source:** CDC. ^*^ Seven travelers were in transit and did not stay in the United States.

**TABLE 1:** Travelers (N = 1,993) arriving from Guinea, Liberia, and Sierra Leone who were screened for Ebola at U.S. airports and their disposition — October 11–November 10, 2014

Port of entry	No. of passengers screened by customs and border protection officers*	Passengers screened by CDC† No. (%)	Disposition after CDC screening (n = 86)		

			No. referred by CDC for medical evaluation§	No. referred by CDC for coordinated disposition with state and local health departments¶	Passengers released to continue travel No. (%)
New York (JFK)	936	26 (2.8)	0	2	24 (92.3)
Washington (IAD)	507	27 (5.3)	3	6	18 (66.7)
Newark (EWR)	204	13 (6.4)	2	0	11 (84.6)
Atlanta (ATL)	136	14 (10.3)	0	1	13 (92.9)
Chicago (ORD)	132	6 (4.5)	2	0	4 (66.7)
Other**	78	0 (—)	0	0	0 (—)
**Total**	**1,993**	**86 (4.3)**	**7**	**9**	**70 (81.4)**

* U.S. Customs and Border Protection officers.

† CDC public health officers screen all travelers identified by customs and border protection officers as potentially having risk for exposure to Ebola or signs or symptoms of Ebola.

§ CDC refers all travelers for medical evaluation who meet the clinical criteria defined in CDC’s Interim U.S. Guidance for Monitoring and Movement of Persons with Potential Ebola Virus Exposure.

¶ The CDC quarantine station coordinates disposition with state and local health departments for travelers who do not meet the clinical criteria for referral for medical evaluation but are categorized as having 1) some risk for exposure to Ebola, or 2) in special circumstances, low (but not zero) risk for exposure.

** Includes travelers who arrived via Anchorage (ANC), Detroit (DTW), Houston (IAH), Los Angeles (LAX), Miami (MIA), Minneapolis/St. Paul (MSP), Montreal (YUL), Ottawa (YOW), Philadelphia (PHL), and Raleigh-Durham (RDU).

**TABLE 2:** Assessment of risk for Ebola among travelers arriving from Guinea, Liberia, and Sierra Leone who were screened by CDC at U.S. airports, by risk category and period — October 11–November 10, 2014

Period	Risk category*			Total

	High	Some	Low (but not zero)	
October 11–17	0	3	11	14
October 18–24	0	6	22	28
October 25–31	0	2	20	22
November 1–7	0	1	13	14
November 8–10†	0	4	4	8
**Total**	**0**	**16**	**70**	**86**

* Guidelines for categorizing risk for Ebola are available at http://www.cdc.gov/vhf/ebola/exposure/risk-factors-when-evaluating-person-for-exposure.html.

† Partial week.

## Footnotes

* Available at http://wwwnc.cdc.gov/travel/page/ebola-outbreak-communication-resources.

† With active monitoring a state or local public health authority assumes responsibility for establishing regular communication with persons whom were possibly exposed to Ebola, including checking daily for symptoms and fever. With direct active monitoring the public health authority conducts active monitoring through direct observation.

§ Additional information on the use of thermometers for screening is available at http://wwwnc.cdc.gov/travel/pdf/ebola-non-contact-temperature-measurement-guidance.pdf.
